# Predicting treatment response to neoadjuvant chemotherapy in locally advanced rectal cancer: A combined deep learning and machine learning approach utilizing longitudinal multi-sequence MRI

**DOI:** 10.1016/j.ejro.2026.100739

**Published:** 2026-02-19

**Authors:** Wengang Zhang, Xiaomei Fu, Li Wen, Yan Yang, Dong Zhang

**Affiliations:** Department of Radiology, XinQiao Hospital, Army Medical University, ChongQing 400037, People's Republic of China

**Keywords:** Rectal cancer, Neoadjuvant chemotherapy, Treatment response, Deep learning, MRI

## Abstract

**Purpose:**

To develop and validate deep leaning-based machine learning models using longitudinal multi-sequence MRI for predicting treatment response of patients with locally advanced rectal cancer (LARC) to neoadjuvant chemotherapy (NCT).

**Methods:**

This retrospective study included 169 LARC patients who received 2–4 cycles of CAPOX chemotherapy before surgery. Patients were randomly divided into a training cohort (n = 118) and a test cohort (n = 51). High-resolution paired MRI sequences (CE-T1WI, T2WI, DWI) were acquired before and after NCT. These sequences were then independently input into a DenseNet121 network to generate predictive probability scores, which served as deep leaning (DL) signatures. Prediction models were built using an SVM classifier. These models were built either by integrating the deep learning signatures alone or by combining them with clinical and radiological features. Model performance was assessed using AUC, accuracy, sensitivity, and specificity. Calibration was evaluated with calibration plots and Brier scores, and clinical utility was analyzed via decision curve analysis (DCA).

**Results:**

In the test cohort, the fusion DL model, integrating pre- and post-NCT multi-sequence DL signatures, achieved an AUC of 0.846. The combined clinical-radiological-deep learning (CRD) model, which added clinical-radiological features to the fusion DL model, reached the highest AUC of 0.851, but the improvement was not statistically significant.

**Conclusions:**

The fusion DL model showed strong performance in predicting pathological response in LARC. The post_DWI signature was the main contributor to the model.

## Highlights

• First study to predict LARC response after neoadjuvant chemotherapy using longitudinal multi-sequence MRI deep learning.
• Longitudinal fusion of pre-&-post multi-sequence MRI lifts AUC to 0.846 without extra clinical data.
• SHAP analysis confirms post-NCT DWI DL-signature as the strongest biomarker of favorable pathological response.
• Adding post-CRM & maximum wall thickness yields peak AUC 0.851, but gain is not statistically significant.

## 1 Introduction

Colorectal cancer (CRC) remains a major global health burden, where rectal cancer accounts for approximately 39 % of new CRC cases and 48 % of CRC-related deaths [1]. Growing research efforts are refining staging systems and enabling risk-stratified precision management for rectal cancer, which facilitates tailored interventions that optimize survival outcomes while preserving patients’ quality of life [2]. For patients with locally advanced rectal cancer (LARC), radical surgery following neoadjuvant therapy (NAT) remains the standard care for high-risk tumors, primarily aimed at reducing local recurrence [3]. NAT downstages more than 50 % of rectal cancers and achieves pathological complete response (pCR) in 10–27 % of patients, with the extent of response being a strong predictor of long-term oncological outcomes [4], [5], [6]. Total neoadjuvant therapy can further increases pCR rates to over 30 %, creating opportunities for organ-preservation strategies [6], [7]. Studies have demonstrated that in MRI-defined low-risk subgroups, a selective radiotherapy-sparing approach using neoadjuvant chemotherapy (NCT) maintains adequate local control while reducing perioperative complications [8], [9]. Tumor heterogeneity and variations in NAT protocols have led to substantial inter-patient variability in treatment response [10], underscoring the critical need for accurate, individualized prediction tools to support truly personalized management.

Medical Imaging techniques, especially high-resolution magnetic resonance imaging (MRI), play a critical role in the precise diagnosis, initial staging, and subsequent restaging of rectal cancer [11], and the combined use of T2-weighted imaging (T2WI), contrast-enhanced T1-weighted imaging (CE-T1WI) and diffusion-weighted imaging (DWI) sequences is indispensable [12]. Morphological and quantitative radiological features driven from these MRI sequences, such as MRI-defined circumferential resection margin (CRM) and distance from the tumor to the anal verge (DTAV), were utilized to predict tumor treatment response [13], [14].

Deep learning (DL) is rapidly emerging as a disruptive tool in medical imaging [15], especially those developed for MRI data, which demonstrating immense potential in identifying subtle radiological features that may indicate tumor treatment response after NAT [16]. Numerous studies have validated the utility of deep learning (DL) in predicting rectal cancer treatment response using either pre- or post-treatment MRI alone [17], [18], [19], [20], [21], [22]. However, such single-time-point analyses inevitably failed to capture therapy-induced biological alterations. This limitation has motivated the integration of multi-time-point imaging data to uncover dynamic tumor changes throughout neoadjuvant therapy.

To our knowledge, no prior study has yet leveraged multi-sequence MRI acquired before and after NCT to predict tumor treatment response. Therefore, this study aims to develop and validate longitudinal multi-sequence MRI-based deep learning models using the DenseNet121 architecture to predict treatment response via tumor regression grade (TRG; TRG 0 and TRG 1 vs TRG 2 and TRG 3) in patients with locally advanced rectal cancer who received NCT alone, with the expectation of providing a more accurate assessment of therapy response.

## 2 Materials and methods

### 2.1 Participants

**Fig. 1:**
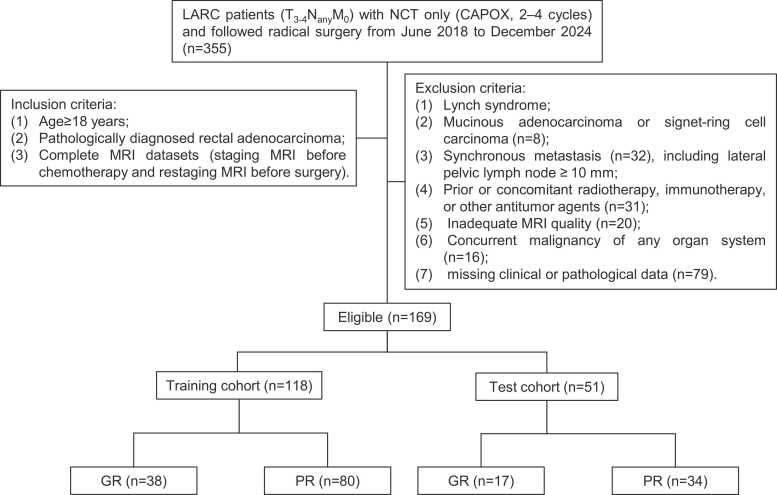
Patient recruitment flow chart. CAPOX, capecitabine plus oxaliplatin; GR, pathological confirmed good response; LARC, locally advanced rectal cancer; MRI, magnetic resonance imaging; NCT, neoadjuvant chemotherapy; PR, pathological confirmed poor response.

This retrospective study was approved by the Ethics Committee of our hospital review board and informed consent was formally waived. A total of 355 patients with locally advanced rectal cancer who received neoadjuvant chemotherapy alone (CAPOX, 2–4 cycles) and subsequently underwent radical surgery at our hospital between June 2018 and December 2024 were included in the study. The inclusion criteria were as follows: (1) age ≥ 18 years; (2) pathologically confirmed rectal adenocarcinoma; (3) availability of complete MRI datasets acquired both before and after chemotherapy. Exclusion criteria included: (1) lynch syndrome (2) mucinous adenocarcinoma or signet-ring cell carcinoma; (3) presence of metastasis (including lateral pelvic lymph node ≥ 10 mm); (4) prior or concomitant radiotherapy, immunotherapy, or treatment with other antitumor agents; (5) poor-quality or incomplete MRI data; (6) concurrent malignancy in any organ system; (7) incomplete clinical or pathological records. As a result, a total of 169 participants were ultimately enrolled and randomly assigned to the training (n = 118) and test cohort (n = 51) in a 7:3 ratio. The patient recruitment process is shown in Fig. 1.

The clinical features, including sex, age, baseline serum biomarkers (CA19–9 and CEA) were obtained from electronic medical records. The neoadjuvant treatment plan was determined by our multidisciplinary team after a comprehensive assessment of the patient's physical condition, baseline tumor stage, expected tumor regression, and predicted prognosis. This study enrolled patients with locally advanced rectal cancer who received neoadjuvant chemotherapy, comprising two to four cycles of CAPOX, prior to radical resection surgery.

### 2.2 Pathologic assessment of response

The pathologic response to neoadjuvant chemotherapy (NCT) was independently evaluated by an experienced gastrointestinal pathologist who remained blinded to all clinical and imaging data, in accordance with the American Joint Committee on Cancer (AJCC) tumor regression grade (TRG) system. The pathological response was categorized using a four-tier scale: TRG 0 (complete response, no viable tumor cells), TRG 1 (near-complete response, rare residual tumor cells), TRG 2 (partial response, residual tumor outgrown by fibrosis), and TRG 3 (minimal or no response, abundant residual tumor) [23]. In this study, TRG 0 and TRG 1 were considered good response (GR), whereas TRG 2 and TRG 3 were considered poor response (PR).

### 2.3 MRI acquisition and feature evaluation

MRI were performed using a 3.0 T system (Discovery MR 750, GE Healthcare, Waukesha, WI) equipped with an eight-channel phased-array body coil, with patients positioned in the supine position. Baseline MRI was obtained a median of 4 days (range, 1–7 days) prior to initiation of neoadjuvant chemotherapy. Post-treatment MRI was performed at a median of 28 days (range, 21–47 days) after completion of all neoadjuvant chemotherapy cycles and within 1 week before radical surgery. This imaging schedule ensured timely pretreatment assessment while allowing adequate evaluation of treatment response prior to surgery [24], [25]. To minimize rectal motility, patients received a 10 mg intramuscular injection of raceanisodamine hydrochloride 20–30 min before undergoing imaging. The imaging protocol included oblique axial, coronal, and sagittal T2-weighted imaging (T2WI); oblique axial diffusion-weighted imaging (DWI); and oblique axial, coronal, and sagittal contrast enhanced T1-weighted imaging (CE-T1WI). CE-T1WI was acquired immediately following the administration of a standard dose (0.1 mmol/kg) of gadopentetate dimeglumine (Kangchen, Guangzhou, China). The scanning volume covered the entire lesion, as confirmed by an abdominal radiologist, with the oblique axial plane-oriented perpendicular to the long axis of the affected bowel segment. Detailed imaging parameters are provided in Table A.1.

The radiological features were evaluated qualitatively and quantitatively in MRI dataset by a board-certified abdominal radiologist (with more than 20 years of experience), who was blinded to all demographic, clinical, and histopathological data. The assessed features, measured both before and after neoadjuvant chemotherapy (NCT), encompassed the longest diameter (LD), maximum wall thickness (MWT), distance from the tumor to the anal verge (DTAV), MRI-defined tumor stage (T stage), MRI-defined nodal stage (N stage), circumferential resection margin (CRM) status, and extramural vascular invasion (EMVI) status.

### 2.4 Image preprocessing and tumor segmentation

Prior to tumor segmentation, the oblique axial MRI sequences (CE-T1WI, T2WI, and DWI) underwent a standardized preprocessing pipeline, which included N4 bias field correction to reduce low-frequency intensity inhomogeneities, image discretization for noise suppression, and intensity normalization to the 0–255 range to minimize quantization artifacts. Tumor segmentation was performed using ITK-SNAP software (v3.8.0). An experienced abdominal radiologist manually delineated the three-dimensional volume of interest (VOI) encompassing the entire tumor. The initial segmentation was then independently reviewed and validated by a senior radiologist with more than 15 years of experience in medical imaging. Using the Onekey tool [26], the two-dimensional region of interest (ROI) corresponding to the maximum cross-sectional area of the tumor was automatically extracted from each segmented VOI across all three sequences (CE-T1WI, T2WI, and DWI). This extraction was performed by computing the tumor area on each axial slice within the VOI and objectively selecting the slice with the largest area, ensuring consistent and reproducible determination of the maximal cross-sectional slice for subsequent deep learning analysis.

### 2.5 Deep learning analysis and signatures development

The deep learning analysis was trained using a DenseNet121 architecture. For each patient, the axial slice displaying the largest cross-sectional area of the rectal tumor was selected from each of the three MRI sequences (CE-T1WI, T2WI, and DWI). All selected images were preprocessed by resampling to a uniform resolution of 256 × 256 pixels. Data augmentation, including random cropping (scale 0.8–1.0, ratio 0.9–1.1) and horizontal flipping (*p* = 0.5), were applied. The model was trained using stochastic gradient descent (SGD) with a learning rate of 0.01 and a batch size of 32. The model’s performance was monitored through the loss function during the entire training process. The final output probability was defined as the deep learning (DL) signature for subsequent analysis. To visualize the image regions that were most critical for the deep learning analysis, gradient-weighted class activation mapping (Grad-CAM) was applied to the final convolutional layer.

### 2.6 Model development and evaluation

We initially screened all clinical and radiological features through univariate logistic regression (*p* < 0.10). Subsequently, we included those features that achieved statistical significance into a multivariate model (*p* < 0.05) to identify independent predictors. These predictors were then integrated to construct a conventional clinical-radiological model.

By utilizing DL signatures acquired both before and after NCT, we first calculated delta signatures. This was obtained by subtracting the post-NCT signatures from the pre-NCT signatures to quantify the longitudinal change.

Based on the pre-NCT, post-NCT, and delta signatures of diverse sequences, support vector machine (SVM) classification models were respectively constructed in the forms of single, pairwise combination, or full fusion. Five-fold cross-validation was employed to optimize and adjust the regularization parameter C and kernel width γ.

**Fig. 2:**
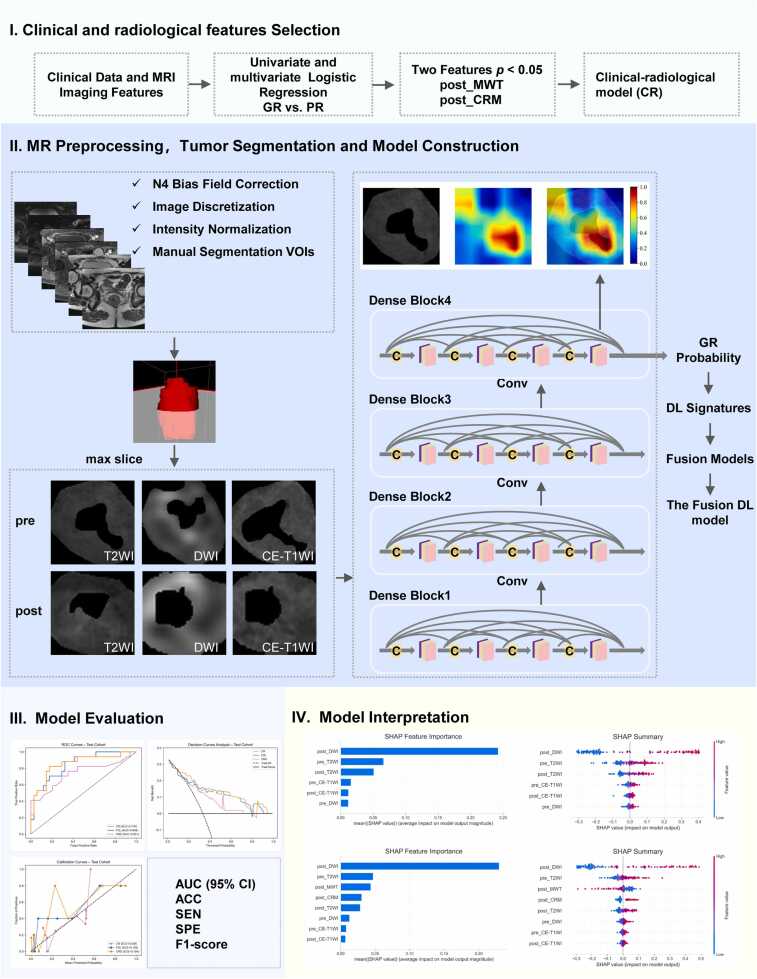
The workflow of this study.

The predictive performance of the models was assessed from three aspects: discrimination, calibration, and clinical utility. Specifically, receiver operating characteristic (ROC) curve analysis, calibration curve evaluation (including Brier scores), and decision curve analysis (DCA) were performed. The evaluation metrics encompassed the area under the curve (AUC) with corresponding 95 % confidence intervals (CI), accuracy, sensitivity, specificity, and the F1-score. The DeLong test was applied to compare AUC values across models. Additionally, SHAP (SHapley Additive exPlanations) analysis was employed to interpret model predictions and identify the most significant contributing features. Fig. 2 illustrates the overall workflow of this study.

### 2.7 Statistical analysis and computational environment

Categorical variables were summarized using frequencies and percentages, expressed as n (%). Group comparisons for categorical variables were conducted using the Chi-square test or Fisher’s exact test, as appropriate. Continuous variables were presented as mean ± standard deviation or median [interquartile range], depending on the normality of their distribution. Between-group differences for continuous variables were evaluated using the independent samples *t*-test for normally distributed data and the Mann–Whitney *U* test for non-normally distributed data. All statistical tests were two-sided, and a *p*-value < 0.05 was considered statistically significant (reported to three decimal places). Statistical analyses were performed using the SciPy stats module in Python (version 3.9.23).

All deep-learning analysis were conducted in Python (version 3.9.23). The DenseNet121 backbone was implemented with PyTorch (version 2.8.0) and torchvision (version 0.23.0). Data preprocessing, augmentation and Grad-CAM visualization were performed with Pandas (version 2.0.3), cv2 (version 4.12.0) and sklearn (version 1.6.1). Training was executed on a mobile workstation equipped with an Intel Core i9–14900HX CPU and 64 GB of DDR5–5200 SDRAM. GPU acceleration was provided by an NVIDIA GeForce RTX 4090 Laptop GPU with 16 GB GDDR6 VRDA (driver 32.0.15.6094, CUDA 12.6).

## 3 Results

### 3.1 Clinical-radiological features and deep learning signatures

In the training and test cohorts, the incidence of pathologically confirmed good response (GR) was 32.2 % (38/118) and 33.3 % (17/51), respectively. Baseline demographic and clinical features, presented in Table 1, showed no significant differences between the two cohorts. As shown in Table 1, post_MWT and post_CRM were all significantly different between good response and poor response groups in both cohorts. The results of univariate and multivariate logistic regression analysis also revealed that post_MWT and post_CRM were significantly associated with GR (Table 2). The clinical-radiological model (CR), which incorporated these two variables using an SVM classifier, achieved an AUC of 0.741 (95 % CI: 0.644–0.827) in the training cohort and 0.740 (95 % CI: 0.555–0.893) in the test cohort.

**Table 1:** Summary of patient demographic and clinical-radiological features.

	Training cohort (n = 118)				Test cohort (n = 51)		
	GR (TRG 0 – 1)	PR (TRG 2 – 3)	*p* Value		GR (TRG 0 – 1)	PR (TRG 2 – 3)	*p* Value
Age (years)	57.50(52.00 – 67.00)	58.50(49.50 – 64.75)	0.510^†^		64.50(57.25 – 68.75)	62.00(50.00 – 66.00)	0.208^†^
Sex			0.899^‡^				1.000^‡^
Female	32(40.00 %)	14(36.84 %)			11(32.35 %)	6(35.29 %)	
Male	48(60.00 %)	24(63.16 %)			23(67.65 %)	11(64.71 %)	
Baseline Hb (g/L)	135.16 ± 13.31	136.21 ± 19.23	0.731*		134.09 ± 15.80	139.94 ± 12.49	0.189*
Baseline CA19–9 (U/mL)			0.707^§^				1.000^§^
≥ 37.00	12(15.00 %)	4(10.53 %)			1(2.94 %)	1(5.88 %)	
< 37.00	68(85.00 %)	34(89.47 %)			33(97.06 %)	16(94.12 %)	
Baseline CEA (ng/mL)			0.887^‡^				0.351^§^
≥ 5.00	30(37.50 %)	13(34.21 %)			14(41.18 %)	4(23.53 %)	
< 5.00	50(62.50 %)	25(65.79 %)			20(58.82 %)	13(76.47 %)	
pre_ LD (mm)	48.00(40.75 — 59.25)	45.50(38.00 — 58.00)	0.478^†^		44.50(39.00 — 56.50)	45.00(36.00 — 52.00)	0.960^†^
post_LD (mm)	40.50(29.00 — 50.00)	36.20(29.25 — 43.75)	0.203^†^		36.50(31.50 — 45.00)	33.00(30.00 — 43.00)	0.441^†^
pre_ MWT (mm)	16.00(13.00 — 19.00)	15.00(12.00 — 18.75)	0.657^†^		17.50(15.00 — 20.00)	17.00(13.00 — 21.00)	0.681^†^
post_MWT (mm)	11.30(9.00 — 15.00)	10.00(8.00 — 11.75)	0.002^†^		14.00(10.25 — 15.75)	9.00(7.00 — 12.00)	0.002^†^
pre_ DTAV (mm)	62.00(45.00 — 94.50)	57.50(41.50 — 87.75)	0.760^†^		55.50(49.25 — 72.75)	62.00(41.00 — 82.00)	0.697^†^
post_DTAV (mm)	63.00(45.00 — 92.25)	60.00(45.50 — 85.75)	0.738^†^		63.00(51.25 — 77.75)	64.00(41.00 — 90.00)	0.689^†^
pre_CRM			0.477^§^				0.185^‡^
Positive	66(82.50 %)	34(89.47 %)			29(85.29 %)	11(64.71 %)	
Negative	14(17.50 %)	4(10.53 %)			5(14.71 %)	6(35.29 %)	
post_CRM			0.039^‡^				0.035^‡^
Positive	59(73.75 %)	20(52.63 %)			24(70.59 %)	6(35.29 %)	
Negative	21(26.25 %)	18(47.37 %)			10(29.41 %)	11(64.71 %)	
pre_EMVI			0.838^§^				0.528^§^
Positive	76(95.00 %)	35(92.10 %)			33(97.06 %)	15(88.23 %)	
Negative	4(5.00 %)	3(7.90 %)			1(2.946 %)	2(11.77 %)	
post_EMVI			0.070^‡^				0.021^§^
Positive	71(88.75 %)	28(73.68 %)			32(94.12 %)	11(64.71 %)	
Negative	9(11.25 %)	10(26.32 %)			2(5.88 %)	6(35.29 %)	
pre_T stage			0.944^‡^				0.260^§^
T3	40(50.00 %)	20(52.63 %)			19(55.88 %)	13(76.47 %)	
T4	40(50.00 %)	18(47.37 %)			15(44.12 %)	4(23.53 %)	
post_T stage			0.067^‡^				0.005^‡^
T2	3(3.75 %)	5(13.16 %)			1(2.94 %)	6(35.29 %)	
T3	48(60.00 %)	25(65.79 %)			19(55.88 %)	8(47.06 %)	
T4	29(36.25 %)	8(21.05 %)			14(41.18 %)	3(17.65 %)	
pre_N stage			0.591^‡^				0.358^‡^
N0	3(3.750 %)	2(5.26 %)			0(0.00 %)	1(5.88 %)	
N1	21(26.25 %)	13(34.21 %)			9(26.47 %)	4(23.53 %)	
N2	56(70.00 %)	23(60.53 %)			25(73.53 %)	12(70.59 %)	
post_N stage			0.061^‡^				0.257^‡^
N0	19(23.75 %)	16(42.10 %)			10(29.41 %)	9(52.94 %)	
N1	38(47.50 %)	17(44.74 %)			14(41.18 %)	5(29.41 %)	
N2	23(28.75 %)	5(13.16 %)			10(29.41 %)	3(17.65 %)	

**Abbreviations:** CRM, MRI-defined circumferential resection margin status; DTAV, distance from tumor to anal verge; EMVI, extramural vascular invasion; GR, good response; LD, longest diameter; MWT, maximal wall thickness; PR, poor response; TRG, tumor regression grade.

**Prefixes:** “pre_” = before neoadjuvant chemotherapy; “post_” = after neoadjuvant chemotherapy.

* *t*-test

† Mann-Whitney *U* test

‡ Chi-square test

§ Fisher's exact test

**Table 2:** Univariate and multivariate regression analysis of clinical and radiological features for good response.

Features	Univariate analysis			Multivariate analysis	
	OR (95 % CI)	*p* Value		OR (95 % CI)	*p* Value
Age (years)	0.987(0.948–1.027)	0.506			
Sex	1.143(0.515–2.535)	0.742			
Baseline Hb (g/L)	1.004(0.979–1.030)	0.729			
Baseline CA19–9 (U/mL)	1.500(0.450–5.001)	0.509			
Baseline CEA (ng/mL)	1.154(0.514–2.590)	0.729			
pre_LD (mm)	0.994(0.971–1.017)	0.589			
post_LD (mm)	0.983(0.957–1.009)	0.204			
pre_MWT (mm)	1.015(0.986–1.045)	0.311			
post_MWT (mm)	0.825(0.724–0.940)	0.004*		0.861(0.755–0.982)	0.026*
pre_DTAV (mm)	0.998(0.986–1.010)	0.732			
post_DTAV (mm)	0.996(0.985–1.008)	0.558			
pre_CRM	0.555(0.169–1.815)	0.330			
post_CRM	2.529(1.127–5.675)	0.025*		1.476(1.053–3.938)	0.036*
pre_EMVI	1.629(0.346–7.670)	0.537			
post_EMVI	2.817(1.035–7.667)	0.043*		1.431(0.454–4.513)	0.541
pre_T stage	0.900(0.415–1.950)	0.789			
post_T stage	0.450(0.218–0.931)	0.031*		0.671(0.296–1.523)	0.340
pre_N stage	0.716(0.367–1.394)	0.326			
post_N stage	0.512(0.291–0.901)	0.020*		0.661(0.352–1.241)	0.197

**Abbreviations:** CRM, MRI-defined circumferential resection margin status (negative = 1, positive = 0); DTAV, distance from tumor to anal verge; EMVI, extramural vascular invasion (negative = 1, positive = 0); LD, longest diameter; MWT, maximal wall thickness; OR, odds ratio; CI, confidence interval.

**Prefixes:** “pre_” = before neoadjuvant chemotherapy; “post_” = after neoadjuvant chemotherapy. * *p*-value < 0.05

**Fig. 3:**
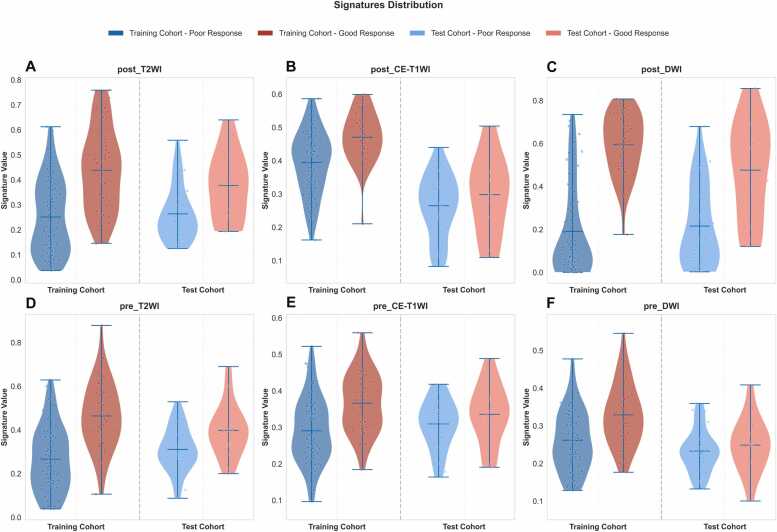
Distribution of six deep learning signatures between training and test cohorts. CE-T1WI, contrast enhanced T1-weighted imaging; DWI, diffusion-weighted imaging; post, after neoadjuvant chemotherapy; pre, before neoadjuvant chemotherapy; T2WI, T2-weighted imaging.

The distribution of deep learning (DL) signatures is shown in Fig. 3. There was a substantial difference in DL signatures between the good response (GR) group and poor response (PR) group (all *p* < 0.001, Table A.2).

### 3.2 Performance of prediction models

The predictive performance of SVM models based on single-sequence DL signatures, assessed by AUC, 95 % CI of AUC, accuracy, sensitivity, and specificity, is summarized in Table A.3. In the test cohort, the SVM model based on deep learning signatures derived from pre- and post-NCT T2WI achieved the highest AUC value of 0.817 (95 % CI: 0.677–0.933), which was comparable to the AUC of 0.803 (95 % CI: 0.654–0.922) obtained by incorporating pre-NCT, post-NCT, and delta T2WI DL signatures. Similarly, the SVM models based on DL signatures derived from DWI exhibited stable and favorable performance, achieving an AUC of 0.792 (95 % CI: 0.658–0.912) for the combination of pre- and post-NCT DWI signatures, and an AUC of 0.806 (95 % CI: 0.678–0.920) for the integration of pre-NCT, post-NCT, and delta DWI deep learning signatures. By contrast, SVM models built on CE‑T1WI deep learning signatures showed comparatively poorer predictive performance, attaining a maximum AUC of 0.696 (95 % CI: 0.538–0.835) with the pre‑ and post‑NCT CE‑T1WI signature combination.

Comprehensive predictive performance metrics of SVM models based on multi-sequence DL signatures are presented in Table A.4. Among models based on pre-NCT signatures, the SVM model integrating pre-NCT T2WI, DWI and CE-T1WI DL signatures demonstrated a marginal improvement in AUC in the test cohort, compared with any SVM model built on pairwise sequence combinations. However, the AUC difference was no statistically significant among all models (DeLong test, all *p* > 0.05). For models founded on post - NCT DL signatures, the SVM model that incorporates all three DL signatures attained AUC values that were statistically indistinguishable from those of any two-signature combination in the test cohort (DeLong test, all *p* > 0.05).

Among all models evaluated, the fused deep learning (DL) model, which integrated all six pre- and post-NCT DL signatures, achieved the highest predictive performance for growth response (GR) in the test cohort, with an AUC of 0.846 (95 % CI: 0.724–0.939). This finding underscores the complementary value of longitudinal DL signatures in capturing tumor response dynamics.

**Fig. 4:**
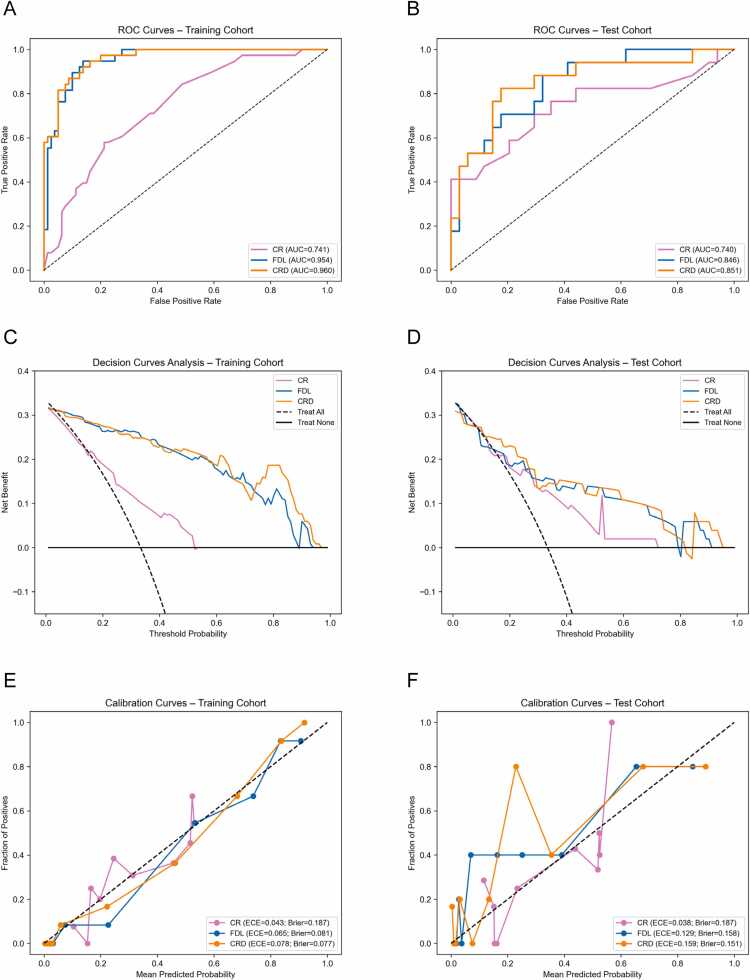
Performance of combination model. The receiver operating characteristic (ROC) curves (A, B), Calibration curves (C, D) and Decision Curve Analysis (DCA) curves (E, F) for the combination models in the training and test cohorts. CR, clinical-radiological model; CRD, combined clinical-radiological-deep learning (CRD) model; ECE, expected calibration error; FDL, multi-sequence fusion deep learning model.

Furthermore, a combined model (CRD) was developed by integrating two clinical-radiological features (post_MWT and post_CRM) into the fusion DL model. The performance of three combined models is summarized in Table 3**.** In the test cohort, the CRD model achieved an AUC of 0.851 (95 % CI: 0.717–0.953), demonstrating significantly better performance than the clinical-radiological model (CR), but no statistically significant improvement was observed compared to the fusion DL model alone(Table A.5). The ROC curves, calibration plot (including Brier scores), and DCA curves are presented in Fig. 4.

**Table 3:** Performance of combined models.

Models	Cohort	Accuracy	AUC (95 % CI)	Sensitivity	Specificity	F1-score
CR	Training	0.720	0.741 (0.644 — 0.827)	0.579	0.788	0.571
	Test	0.706	0.740 (0.555 — 0.893)	0.706	0.706	0.615
FDL	Training	0.890	0.954 (0.918 — 0.982)	0.947	0.863	0.847
	Test	0.745	0.846 (0.724 — 0.939)	0.882	0.677	0.698
CRD	Training	0.873	0.960 (0.926 — 0.984)	0.947	0.838	0.828
	Test	0.824	0.851 (0.717 — 0.953)	0.824	0.824	0.757

**Abbreviations:** AUC, area under the curve; CI, confidence interval; CR, clinical-radiological model; CRD, a clinical-radiological deep learning combination model; FDL, fusion deep learning model.

### 3.3 Model interpretation

**Fig. 5:**
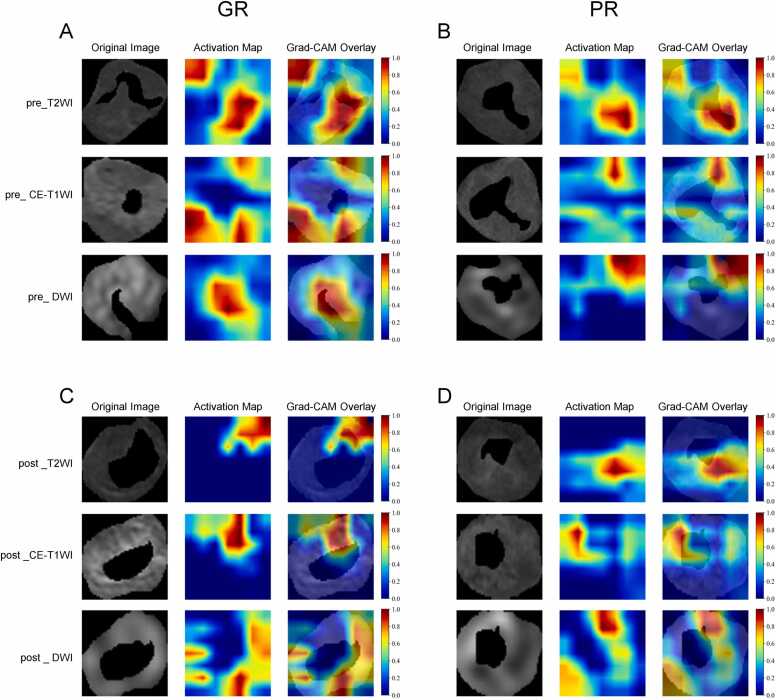
Gradient-weighted Class Activation Mapping (Grad-CAM) visualization of deep learning model attention. (A, C) A 47-year-old woman with locally advanced rectal cancer who achieved a pathologically confirmed good response (GR). (B, D) A 63-year-old woman with locally advanced rectal cancer who had a pathologically confirmed poor response (PR).

**Fig. 6:**
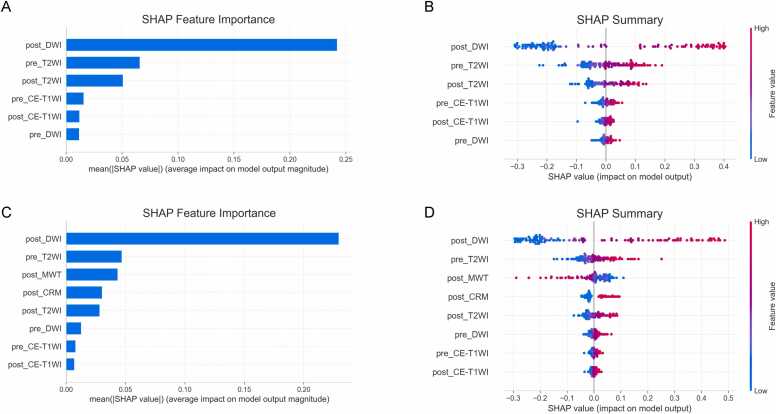
Shapley Additive Explanations (SHAP) analysis of fusion DL and CRD models. (A, C) Global feature importance rankings based on mean absolute SHAP values for predicting good response (GR). (B, D) Beeswarm plots show how each feature affects the predicted probability of GR. Positive SHAP values (x-axis) push the prediction toward GR; negative values pull it away. Red dots indicate high feature values; blue dots indicate low values. SVM, support vector machine.

Grad-CAM heatmaps confirmed that the networks focused their decision-making process on the intratumoral region. Representative Grad-CAM visualizations for one GR and one PR patient are shown in Fig. 5. SHAP analysis was performed to interpret the prediction mechanisms of both the fused DL model and the CRD model. As illustrated in the feature importance plot (Figs. 6A, 6C), the post_DWI signature emerged as the most influential predictor in both models, exhibiting the highest mean absolute SHAP value. This finding indicates that DWI acquired after NCT contributes most substantially to predicting GR. The SHAP summary plot (Figs. 6B, 6D) provides further insight into models by illustrating the magnitude and directionality of each feature’s contribution. Specifically, higher values of post_DWI signature (denoted in red) are associated with positive SHAP values, indicating a positive association with the probability of GR. The pre_T2WI and post_MWT signature ranked second and third in feature importance, respectively.

## 4 Discussion

This study developed and validated a machine-learning model that utilizes deep learning signatures extracted from longitudinal multi-sequence MRI to predict the tumor treatment response in patients with locally advanced rectal cancer (LARC) after neoadjuvant chemotherapy (NCT). The fusion DL model, which integrated longitudinal multi-sequence data, outperformed all single-time-point single sequence models, with the post_DWI signature being the most influential factor contributing to the model’s predictions. To the best of our knowledge, this is the first study to predict pathological tumor treatment response using longitudinal multi-sequence MRI in patients with LARC after NCT.

Of the clinical-radiological features evaluated, post-treatment circumferential resection margin (CRM) status and maximum wall thickness (MWT) were identified as independent predictors. However, integrating them into the fusion DL model to construct a clinical-radiological-deep learning (CRD) model did not yield a significant performance improvement. This suggests that these clinical-radiological features do not provide substantial additional predictive value within the existing deep learning framework.

Some studies [13], [27] have reported varying outcomes regarding clinical and imaging predictors of treatment response in LARC. Nonetheless, uninvolved CRM has consistently been recognized as a reliable indicator of favorable pathological response [13], [28], [29] which is consistent with our findings. Notably, this study is the first to identify an association between preoperative MWT and tumor response, indicating that a smaller preoperative MWT is associated with a higher probability of favorable pathological response. Furthermore, when comparing the paired radiological features before and after treatment, all features, with the exception of distance from the tumor to the anal verge (DTAV) in the good response group, exhibited statistically significant differences (Table A.6). The lack of a significant difference in DTAV may be due to the relatively small sample size of the favorable response subgroup, which reduced the statistical power to detect subtle changes. In addition, the absence of a significant association between baseline MWT or CRM status and treatment response suggests that these pre-NCT MRI features may reflect initial tumor burden rather than capturing the dynamic biological changes induced by treatment.

In this study, we implemented the DenseNet121 architecture as our deep learning backbone. Its dense connectivity promotes feature reuse and helps mitigate the gradient vanishing problem, which is particularly advantageous when training with limited datasets [30]. Grad-CAM visualizations confirmed that DenseNet121 correctly localized attention to intratumoral regions, validating its decision-making processes. This aligns with the fundamental requirement for interpretable artificial intelligence in clinical practice, as reliable lesion identification ensures DenseNet121 predictions are based on relevant pathological tissue rather than extratumoral noise.

While most previous studies predicting LARC response to neoadjuvant therapy have relied on single-sequence or single-time-point MRI data [31], [32], our study provides a comprehensive, longitudinal assessment by systematically evaluating the combined value of multi-sequence MRI acquired both before and after NCT. A key finding is that the post_DWI signature was the most important predictor of good response, underscoring the pivotal role of post-NCT DWI in evaluating tumor regression. This result aligns with the findings of Pham et al., [33] who demonstrated that DWI after neoadjuvant therapy effectively captured NCT-induced alterations in tumor cellularity. Notably, the improved predictive performance achieved by combining DWI and T2WI further corroborates prior research, including a recent investigation showing that integrating DWI with T2WI improves the accuracy of tumor regression grading in LARC following NCT [34]. Consistent with this, Jang et al. [35] emphasized that DWI should serve as a complement to T2WI. In contrast, the signature based on CE-T1WI exhibited inferior performance and failed to confer substantial added value when incorporated into the fusion model. Prior investigations have yielded analogous findings. Zhang et al. [36] reported that the CE-T1WI modality achieved the lowest sensitivity, indicating that CE-T1WI-derived features do not substantially contribute to improving predictive performance.

Meanwhile, the longitudinal fusion models of each sequence (e.g., T2WI and CE-T1WI) all demonstrated improved predictive performance compared to their single-time-point counterparts, indicating that the integration of temporal data may contribute to enhanced prediction accuracy. This observation may partly explain the high tumor response prediction accuracy of systems like DeepRP-RC [37]. However, incorporating delta signatures (e.g., pre+post+delta_T2WI) did not confer any additional predictive benefit. In our study, the combination of all DL signatures from both pre- and post-NCT and two radiological features showed significant difference compared to the clinical-radiological model (CR), and SHAP analysis suggesting post-NCT DWI signal intensity is the strongest predictor of good response, with pre_T2WI and post_MWT offering complementary and interpretable information. This result supports the adoption of a multi-sequence, longitudinal MRI protocol for routine staging, restaging and surveillance in LARC.

Models based on delta signatures and CE - T1WI signatures demonstrated limited incremental value in our study. From a technical perspective, our CE-T1WI scanning protocol was initiated immediately following contrast administration, ensuring consistent timing across all participants. While this approach removes variability caused by injection-to-scan delays, it limits all lesions to the earliest enhancement phase. As a result, the dynamic vascular permeability kinetics that evolve rapidly after contrast administration are confined to a single time point, which reduces the additional discriminatory power of CE-T1WI. Future studies may employ multiphase T1WI or high-temporal-resolution dynamic contrast-enhanced (DCE) imaging to further evaluate whether vascular permeability dynamics provide incremental diagnostic value. Additionally, as pairwise rigid registration was not performed, residual voxel displacement may have introduced partial-volume fluctuations, potentially obscuring true microenvironmental changes and further diminishing the incremental utility of delta signatures. Moreover, with a median interval of 28 days (range: 21–47 days) between the last chemotherapy cycle and preoperative MRI, the tumor bed is likely to contain a complex mixture of fibrosis, necrosis, and inflammatory infiltrates. These pathological components may exert opposing effects on signal intensity, potentially neutralizing each other and resulting in no appreciable net change in signal. This phenomenon may explain the limited predictive value of delta-signatures observed in our cohort.

From a clinical perspective, this predictive model may act as a complementary tool for preoperative risk stratification following further validation and optimization. It could assist clinical decision-making by noninvasively predicting treatment response. Good responses may support organ-preservation or minimally invasive surgery.

Our study has several limitations. First, its single-center, retrospective design and modest sample size necessitate external validation before broader application. Second, Tumor segmentation was performed manually on rectal MRI, as auto-segmentation remains immature for this anatomy. Third, model input was restricted to the single largest axial tumor slice. Although this 2D strategy yielded acceptable performance under the present 4-mm-thick MRI protocol, it inevitably discards volumetric heterogeneity. Fourth, the cohort was restricted to patients who received two to four cycles of CAPOX, which limits generalizability to other regimens. However, this shorter treatment regimen reduced cumulative toxicity and allowed earlier identification of poor responders, supporting timely treatment modification or upfront surgery [38].

In conclusion, the integration of longitudinal multi-sequence MRI deep learning signatures with radiological features (post_MWT and post_CRM) provides an accurate, efficient tool for tumor treatment response assessment in LARC after NCT and holds promise for integration into clinical decision support systems. Considering the limitations of our study, future work should prioritize automated segmentation techniques that tolerate multi-sequence misalignment and perform prospective validation to confirm model’s clinical value.

## CRediT authorship contribution statement

**Wengang Zhang:** Writing – original draft, Investigation, Data curation, Conceptualization. **Li Wen:** Supervision, Conceptualization. **Xiaomei Fu:** Visualization, Formal analysis. **Dong Zhang:** Writing – review & editing, Methodology, Conceptualization. **Yan Yang:** Writing – review & editing, Software, Formal analysis.

## Ethical statement

The studies involving human participants were reviewed and approved by the Institutional Review Board of Xinqiao Hospital, Army Medical University (approval number: 2025-YD −088–01). The institutional review board waived the requirement for written informed consent due to the retrospective nature of the study.

## Funding

This research did not receive any specific grant from funding agencies in the public, commercial, or not-for-profit sectors.

## Declaration of Competing Interest

The authors declare that they have no known competing financial interests or personal relationships that could have appeared to influence the work reported in this paper.

## Data Availability

The data that support the findings of this study are available from the corresponding author Dong Zhang upon reasonable request. The data are not publicly available due to privacy concerns.
