# Supplementary material for: Predicting treatment response to neoadjuvant chemotherapy in locally advanced rectal cancer: A combined deep learning and machine learning approach utilizing longitudinal multi-sequence MRI
Source: Eur J Radiol Open. 2026 Feb 19;16:100739. doi: 10.1016/j.ejro.2026.100739 (PMC12933455; doi:10.1016/j.ejro.2026.100739)
Supplement: Supplementary file 1 — Supplementary material [file mmc1.docx]

**Table A.1** MR Scan Parameters

| Sequence | TR (ms) | TE (ms) | FOV (cm) | Matrix | Bandwidth (kHz/Px) | NEX | ETL |
| --- | --- | --- | --- | --- | --- | --- | --- |
| oblique axial FSE T2 | 3360 | 103.04 | 20×20 | 512×512 | 195.312 | 2 | 15 |
| oblique axial DWI (b=800) | 4500 | 79.5 | 20×20 | 256×256 | 1953.12 | 8 | 1 |
| oblique axial FSPGR T1 FS +C | 215 | 2.1 | 20×20 | 512×512 | 122.07 | 1.5 | 1 |

**Abbreviations:** FSE, Fast spin echo; DWI, Diffusion weighted imaging; FSPGR, Spoiled gradient recalled echo; FS, Fat Suppression; TR, Repetition time; TE, Echo time; FOV, Field of view; NEX, Number of excitations; ETL, Echo train length.

**Table A.2** Summary of deep learning signatures value

| Signatures | Good Response (GR) | Poor Response (PR) | *p* Value |
| --- | --- | --- | --- |
| post_T2WI | 0.41(0.28-0.51) | 0.23(0.14-0.35) | **0.000**^†^ |
| post_CE-T1WI | 0.44(0.37-0.50) | 0.37(0.27-0.44) | **0.000**^†^ |
| post_DWI | 0.59(0.45-0.70) | 0.10(0.04-0.33) | **0.000**^†^ |
| pre_T2WI | 0.43(0.35-0.54) | 0.27(0.17-0.37) | **0.000**^‡^ |
| pre_CE-T1WI | 0.34(0.31-0.42) | 0.30(0.24-0.35) | **0.000**^‡^ |
| pre_DWI | 0.30(0.25-0.35) | 0.24(0.20-0.31) | **0.000**^†^ |

**Abbreviations:** CE-T1WI, contrast-enhanced T1-weighted imaging; DWI, diffusion-weighted imaging; T2WI, T2-weighted imaging.
**Prefixes:** “pre_” = before neoadjuvant chemotherapy; “post_” = after neoadjuvant chemotherapy.

Note: Values for all deep learning signatures are reported as the median (interquartile range).

‡ t-test

† Mann-Whitney U test

**Table A.3** SVM model performance using single-sequence DL signatures to predict treatment response

| Sequence | Merics | Training cohort / Test cohort | | | | |
| --- | --- | --- | --- | --- | --- | --- |
|  |  | delta | pre+delta | post+delta | pre+post | pre+post+delta |
| T2WI | AUC | 0.638 / 0.561 | 0.859 / 0.756 | 0.872 / 0.791 | 0.874 / 0.817 | 0.884 / 0.803 |
|  | 95% CI^⁕^ | [0.524, 0.735] /  [0.371, 0.742] | [0.788, 0.918] /  [0.594, 0.884] | [0.808, 0.933] /  [0.640, 0.915] | [0.806, 0.936] /  [0.677, 0.933] | [0.818, 0.941] /  [0.654, 0.922] |
|  | ACC | 0.568 / 0.706 | 0.805 / 0.745 | 0.814 / 0.706 | 0.805 / 0.745 | 0.822 / 0.745 |
|  | SEN | 0.842 / 0.471 | 0.842 / 0.824 | 0.790 / 0.941 | 0.974 / 0.941 | 0.868 / 0.941 |
|  | SPE | 0.438 / 0.824 | 0.788 / 0.706 | 0.825 / 0.588 | 0.725 / 0.647 | 0.800 / 0.647 |
| DWI | AUC | 0.871 / 0.775 | 0.901 / 0.796 | 0.894 / 0.803 | 0.918 / 0.792 | 0.908 / 0.806 |
|  | 95% CI^⁕^ | [0.800, 0.931] /  [0.647, 0.898] | [0.844, 0.947] /  [0.668, 0.918] | [0.836, 0.944] /  [0.677, 0.919] | [0.867, 0.959] /  [0.658, 0.912] | [0.853, 0.952] /  [0.678, 0.920] |
|  | ACC | 0.805 / 0.784 | 0.805 / 0.784 | 0.814 / 0.765 | 0.839 / 0.745 | 0.822 / 0.745 |
|  | SEN | 0.947 / 0.765 | 0.974 / 0.765 | 0.974 / 0.706 | 0.974 / 0.706 | 0.974 / 0.706 |
|  | SPE | 0.738 / 0.794 | 0.725 / 0.794 | 0.738 / 0.794 | 0.775 / 0.765 | 0.750 / 0.765 |
| CE-T1WI | AUC | 0.716 / 0.548 | 0.780 / 0.627 | 0.828 / 0.620 | 0.797 / 0.696 | 0.806 / 0.652 |
|  | 95% CI^⁕^ | [0.616, 0.804] /  [0.363, 0.716] | [0.683, 0.864] /  [0.456, 0.792] | [0.746, 0.901] /  [0.436, 0.790] | [0.711, 0.885] /  [0.538, 0.835] | [0.726, 0.887] /  [0.468, 0.812] |
|  | ACC | 0.737 / 0.667 | 0.653 / 0.686 | 0.771 / 0.726 | 0.771 / 0.628 | 0.780 / 0.706 |
|  | SEN | 0.474 / 0.353 | 0.868 / 0.588 | 0.711 / 0.529 | 0.632 / 0.824 | 0.632 / 0.588 |
|  | SPE | 0.863 / 0.824 | 0.550 / 0.735 | 0.800 / 0.824 | 0.838 / 0.529 | 0.850 / 0.765 |

**Abbreviations:** ACC, accuracy; CE-T1WI, contrast-enhanced T1-weighted imaging; delta, the absolute change of (pre – post); DWI, diffusion-weighted imaging; post, post-neoadjuvant chemotherapy; pre, pre-neoadjuvant chemotherapy; SEN, sensitivity; SPE, specificity; T2WI, T2-weighted imaging.

**Note:** The “+” symbol denotes sequences or time-points combination. “95% CI^⁕^” represent the 95% CI of AUC in training cohort / test cohort.

**Table A.4** SVM model performance using multiple-sequence DL signatures to predict treatment response

| Sequence | Merics | Training cohort / Test cohort | | | | | | |
| --- | --- | --- | --- | --- | --- | --- | --- | --- |
|  |  | pre | post | delta | pre+delta | post+delta | pre+post | pre+post+delta |
| T2WI+DWI | AUC | 0.830 / 0.713 | 0.907 / 0.822 | 0.866 / 0.791 | 0.923 / 0.825 | 0.914 / 0.829 | 0.941 / 0.834 | 0.935 / 0.832 |
|  | 95% CI^⁕^ | [0.742, 0.899] /  [0.538, 0.853] | [0.850, 0.953] /  [0.690, 0.931] | [0.798, 0.926] /  [0.666, 0.907] | [0.875, 0.961] /  [0.704, 0.926] | [0.864, 0.956] /  [0.703, 0.932] | [0.898, 0.974] /  [0.714, 0.934] | [0.892, 0.970] /  [0.711, 0.935] |
|  | ACC | 0.814 / 0.765 | 0.848 / 0.784 | 0.822 / 0.784 | 0.839 / 0.765 | 0.831 / 0.765 | 0.881 / 0.726 | 0.864 / 0.824 |
|  | SEN | 0.658 / 0.647 | 0.974 / 0.647 | 0.947 / 0.765 | 0.921 / 0.765 | 0.921 / 0.765 | 0.947 / 0.882 | 0.895 / 0.529 |
|  | SPE | 0.888 / 0.824 | 0.788 / 0.853 | 0.763 / 0.794 | 0.800 / 0.765 | 0.788 / 0.765 | 0.850 / 0.647 | 0.850 / 0.971 |
| T2WI+CE-T1WI | AUC | 0.852 / 0.725 | 0.801 / 0.746 | 0.874 / 0.730 | 0.875 / 0.747 | 0.875 / 0.792 | 0.944 / 0.818 | 0.916 / 0.827 |
|  | 95% CI^⁕^ | [0.772, 0.922] /  [0.552, 0.862] | [0.718, 0.878] /  [0.578, 0.896] | [0.808, 0.933] /  [0.582, 0.870] | [0.806, 0.934] /  [0.578, 0.881] | [0.807, 0.936] /  [0.647, 0.915] | [0.898, 0.980] /  [0.680, 0.938] | [0.860, 0.962] /  [0.694, 0.937] |
|  | ACC | 0.822 / 0.706 | 0.720 / 0.745 | 0.797 / 0.745 | 0.805 / 0.745 | 0.746 / 0.765 | 0.898 / 0.843 | 0.856 / 0.745 |
|  | SEN | 0.816 / 0.765 | 0.816 / 0.647 | 0.947 / 0.765 | 0.868 / 0.824 | 0.947 / 0.647 | 0.842 / 0.765 | 0.921 / 0.882 |
|  | SPE | 0.825 / 0.677 | 0.675 / 0.794 | 0.725 / 0.735 | 0.775 / 0.706 | 0.650 / 0.824 | 0.925 / 0.882 | 0.825 / 0.677 |
| CE-T1WI+DWI | AUC | 0.805 / 0.638 | 0.904 / 0.801 | 0.874 / 0.730 | 0.907 / 0.796 | 0.904 / 0.801 | 0.932 / 0.791 | 0.917 / 0.805 |
|  | 95% CI^⁕^ | [0.715, 0.880] /  [0.470, 0.810] | [0.848, 0.952] /  [0.673, 0.919] | [0.808, 0.933] /  [0.582, 0.870] | [0.850, 0.955] /  [0.658, 0.921] | [0.848, 0.952] /  [0.673, 0.919] | [0.886, 0.969] /  [0.660, 0.913] | [0.865, 0.959] /  [0.678, 0.922] |
|  | ACC | 0.754 / 0.726 | 0.814 / 0.765 | 0.797 / 0.745 | 0.814 / 0.784 | 0.814 / 0.765 | 0.856 / 0.765 | 0.822 / 0.784 |
|  | SEN | 0.737 / 0.471 | 0.974 / 0.765 | 0.947 / 0.765 | 0.974 / 0.706 | 0.974 / 0.765 | 0.974 / 0.706 | 0.974 / 0.706 |
|  | SPE | 0.763 / 0.853 | 0.738 / 0.765 | 0.725 / 0.735 | 0.738 / 0.824 | 0.738 / 0.765 | 0.800 / 0.794 | 0.750 / 0.824 |
| T2WI+CE-T1WI+DWI | AUC | 0.880 / 0.728 | 0.915 / 0.820 | 0.876 / 0.768 | 0.933 / 0.827 | 0.915 / 0.822 | **0.954 / 0.846** | 0.940 / 0.834 |
|  | 95% CI^⁕^ | [0.809, 0.939] /  [0.552, 0.869] | [0.863, 0.959] /  [0.689, 0.935] | [0.815, 0.932] /  [0.633, 0.893] | [0.888, 0.967] /  [0.709, 0.930] | [0.864, 0.956] /  [0.694, 0.933] | **[0.918, 0.982] /  [0.724, 0.939]** | [0.898, 0.972] /  [0.707, 0.939] |
|  | ACC | 0.805 / 0.726 | 0.848 / 0.726 | 0.822 / 0.765 | 0.839 / 0.784 | 0.822 / 0.745 | **0.890 / 0.745** | 0.873 / 0.824 |
|  | SEN | 0.816 / 0.706 | 0.974 / 0.882 | 0.947 / 0.765 | 0.921 / 0.706 | 0.947 / 0.765 | **0.947 / 0.882** | 0.895 / 0.588 |
|  | SPE | 0.800 / 0.735 | 0.788 / 0.647 | 0.763 / 0.765 | 0.800 / 0.824 | 0.763 / 0.735 | **0.863 / 0.677** | 0.863 / 0.941 |

**Abbreviations:** ACC, accuracy; CE-T1WI, contrast-enhanced T1-weighted imaging; delta, the absolute change of (pre – post); DWI, diffusion-weighted imaging; post, post-neoadjuvant chemotherapy; pre, pre-neoadjuvant chemotherapy; SEN, sensitivity; SPE, specificity; T2WI, T2-weighted imaging.

**Note:** The “+” symbol denotes sequences or time-points combination. “95% CI^⁕^” represent the 95% CI of AUC in training cohort / test cohort.

**Table A.5** DeLong test for pairwise AUC differences among all final fusion models in the test cohort

| Comparison | AUC_1 | AUC_2 | Difference | Z | *p* Value |
| --- | --- | --- | --- | --- | --- |
| CR vs FDL | 0.740 | 0.846 | -0.106 | -4.766 | **0.000** |
| CR vs CRD | 0.740 | 0.851 | -0.112 | -6.070 | **0.000** |
| FDL vs CRD | 0.846 | 0.851 | -0.005 | -0.364 | 0.716 |

**Abbreviations:** CR, clinical-radiological model; CRD, combined clinical-radiological-deep learning model; FDL, multi-sequence fusion deep learning model.
**Note:** vs = versus

**Table A.6** Changes in paired radiological characteristics (pre- vs post-treatment)

|  | Good Response (TRG 0 – 1) | | |  | Poor Response (TRG 2 – 3) | | |
| --- | --- | --- | --- | --- | --- | --- | --- |
|  | pre | post | *p* Value |  | pre | post | *p* Value |
| CRM |  |  | 0.000^‡^ |  |  |  | 0.014^‡^ |
| Positive | 45(81.8%) | 26(47.3%) |  |  | 95(83.3%) | 83(72.8%) |  |
| Negative | 10(18.2%) | 29(52.7%) |  |  | 19(16.7%) | 31(27.2%) |  |
| EMVI |  |  | 0.003^‡^ |  |  |  | 0.041^‡^ |
| Positive | 50(90.9% | 39(70.9%) |  |  | 109(95.6%) | 103(90.4%) |  |
| Negative | 5(9.1%) | 16(29.1%) |  |  | 5(4.4%) | 11(9.6%) |  |
| LD (mm) | 45.00 (37.00 – 58.00) | 35.00 (29.50 – 43.50) | 0.000^†^ |  | 47.00 (39.25 – 59.00) | 40.00 (30.00 – 49.75) | 0.000^†^ |
| MWT (mm) | 16.00 (12.00 – 20.00) | 10.00 (7.50 – 12.00) | 0.000^†^ |  | 16.00 (13.00 – 19.00) | 12.00 (10.00 – 15.00) | 0.000^†^ |
| DTAV (mm) | 59.00 (41.00 – 85.00) | 61.00 (44.00 – 88.00) | 0.231^†^ |  | 60.00 (45.25 – 80.00) | 63.00 (48.00 – 87.25) | 0.000^†^ |
| N stage |  |  | 0.000^†^ |  |  |  | 0.000^†^ |
| N0 | 3(5.45%) | 25(45.45%) |  |  | 3(2.63%) | 29(25.44%) |  |
| N1 | 17(30.91%) | 22(40.00%) |  |  | 30(26.32%) | 52(45.61%) |  |
| N2 | 35(63.64%) | 8(14.55%) |  |  | 81(71.05%) | 33(28.95%) |  |
| T stage |  |  | 0.000^†^ |  |  |  | 0.000^†^ |
| T0 | 0(0.00%) | 11(20.00%) |  |  | 0(0.00%) | 4(3.51%) |  |
| T1 | 33(60.00%) | 33(60.00%) |  |  | 59(51.75%) | 67(58.77%) |  |
| T2 | 22(40.00%) | 11(20.00%) |  |  | 55(48.25%) | 43(37.72%) |  |

**Abbreviations:** CRM, MRI-defined circumferential resection margin status; DTAV, distance from tumor to anal verge; EMVI, extramural vascular invasion; LD, longest diameter; MWT, maximal wall thickness; TRG, tumor regression grade.

† Wilcoxon signed-rank test

‡ McNemar's test.
